# De Novo Whole-Genome Sequencing and Assembly of the Yellow-Throated Bunting (*Emberiza elegans*) Provides Insights into Its Evolutionary Adaptation

**DOI:** 10.3390/ani12152004

**Published:** 2022-08-08

**Authors:** Tingli Hu, Guotao Chen, Zhen Xu, Site Luo, Hui Wang, Chunlin Li, Lei Shan, Baowei Zhang

**Affiliations:** 1 School of Life Sciences, Anhui University, Hefei 230601, China; 2 School of Life Sciences, Xiamen University, Xiamen 361102, China; 3 College of Food and Bioengineering, Bengbu University, Bengbu 233030, China; 4 School of Resources and Environmental Engineering, Anhui University, Hefei 230601, China; 5 School of Life Sciences, Nanjing Normal University, Nanjing 210023, China

**Keywords:** adaptation, *Emberiza elegans*, genome, Nanopore sequencing

## Abstract

**Simple Summary:**

We report the genomic sequence of *Emberiza elegans* for understanding the evolutionary mechanisms of environmental adaptation and for studying a more effective genetic monitoring of this species. The *E. elegans* assembly was approximately 1.14 Gb, with a scaffold N50 of 28.94 Mb. About 15,868 protein-coding genes were predicted, and 16.62% of the genome was identified as having repetitive elements. Our genomic evolution analyses found considerable numbers of adaptive genes that may help the yellow-throated bunting cope with migratory behavior and environmental stressors of diseases. These results provide us with new insights into genomic evolution and adaptation, thus providing a valuable resource for further studies of population genetic diversity and genome evolution in this species.

**Abstract:**

Yellow-throated bunting is a small migratory songbird unique to the Palearctic region. However, the genetic studies of this species remain limited, with no nuclear genomic sequence reported to date. In this study, the genomic DNA from the bird was sequenced in long reads using Nanopore sequencing technology. Combining short-read sequencing, the genome was well-assembled and annotated. The final length of the assembly is approximately 1.14 Gb, with a scaffold N50 of 28.94 Mb. About 15,868 protein-coding genes were predicted, and 16.62% of the genome was identified as having repetitive elements. Comparative genomic analysis showed numerous expanded gene families and positively selected genes significantly enriched in those KEGG pathways that are associated with migratory behavior adaptation and immune response. Here, this newly generated de novo genome of the yellow-throated bunting using long reads provide the research community with a valuable resource for further studies of population genetic diversity and genome evolution in this species.

## 1. Introduction

**Figure 1:**
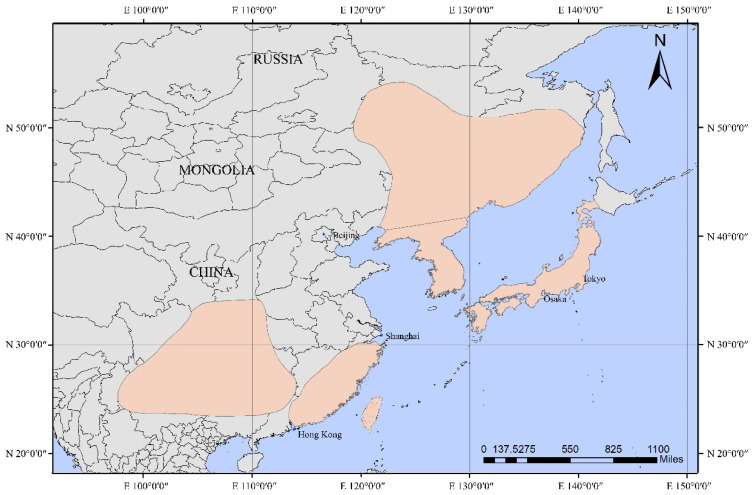
The distribution map of the yellow-throated bunting. The orange area is the marked species distribution. Distribution data were obtained from the IUCN Red List of Threatened Species Version 2018-2. (https://www.iucnredlist.org. Accessed on 5 January 2022.)

The yellow-throated bunting (*E. elegans*, Emberizidae, Passeriformes) is a common songbird, which is widely distributed in Eurasia (Figure 1), and mainly inhabits the foothills, hills, or thickets at lower altitudes [1]. Currently, three subspecies of the yellow-throated buntings are suggested to be in China [2], namely *E. elegans. elegans* (mainly distributed in Taiwan), *E. elegans elegantula* (mainly distributed in Sichuan, Yunnan-Guizhou Plateau), and *E. elegans ticehursti* (mainly distributed throughout eastern China). They normally breed in the high latitudes of the Palearctic region and migrate to lower latitudes for the winter months [3] except for *E. elegans elegantula*, which is usually a resident bird [2]. Like other buntings, the common ancestor of yellow-throated buntings may have originated from the Old World and gained their ancestral migratory ability through long-term evolution [4]. However, it is worth noting that the research on this migratory species has primarily focused on migration patterns [3], phylogenetic relationships [5], reproductive behaviors [6], and other fields. Few studies have been conducted for investigating the genetic mechanisms of its adaption to the environment, historical population sizes, and genetic diversity, which limits our understanding of the yellow-throated bunting.

Comparative genomics is a powerful tool to study gene and genome evolution among various species [7] and provides insights into the mechanisms of species evolution, physiology, morphology and development, genome composition, and how we can use genomic information for bird conservation [8]. However, there have been few comparative genomic studies on yellow-throated bunting, which prevents us from gaining insights into this species from a genomic evolutionary perspective.

To advance research in yellow-throated buntings’ comparative genomics and contribute to the further development of genetic research for this species, we de novo sequenced and assembled the genome of the yellow-throated bunting (*E. elegans ticehursti*). Then, annotation and comparative genomic analyses, including orthology, rapid expansion, and identification of positively selected genes were conducted, which allowed us to deepen our understanding of the evolutionary history and relationships between this and other birds at the molecular level.

## 2. Materials and Methods

### 2.1. Sample Collection and Sequencing

Muscle samples for whole-genome sequencing were collected from a living healthy, adult male *E. elegans* (*E. elegans ticehursti*) individual from Hefei, Anhui, China, and archived as a voucher specimen at the Biological Museum of Anhui University (AHU2021HF0102). Genomic DNA was extracted and isolated from the collected muscle and then used to construct Nanopore libraries, 10× Genomics linked-read libraries, and Illumina short-read libraries. The quality of the genomic DNA was evaluated by using a NanoPhotometer spectrophotometer.

Long-insert Nanopore libraries were constructed using a Ligation Sequencing 1D Kit as recommended by the manufacturer. Genomic DNA was firstly broken into fragments larger than 15 kb, and then fragment sizes were selected with a Blue Pippin System. The single-molecule, real-time sequencing of long reads was conducted on one flow cell of a PromethION platform (Oxford Nanopore Technologies, Oxford, UK).

The linked-read sequencing libraries of 10× Genomics were generated using Chromium™ Genome Reagent Kits v2, according to the manufacturer’s instructions. The resulting 10× Genomics libraries were sequenced with 350 bp size by using an Illumina HiSeq^TM^2000 platform.

### 2.2. Genome Survey and Assembly

To estimate the yellow-throated bunting’s genome size, high-quality short reads (86.04 G) were used to extract the 17-mer sequences by using sliding windows. The frequency of each 17-mer was calculated from the clean reads using the Jellyfish Version 2.3.0 software [9]. Finally, genomic size and heterozygosity ratio were estimated using the GCE [10].

Initially, contigs were de novo assembled with Nanopore reads using the Canu software [11]. Then, the produced contigs were first polished using Racon [12] with default parameters. Subsequently, 10× Genomics data were used to scaffold the contigs, and then the scaffolds were upgraded with the PBjelly [13]. The second round of error correction based on Illumina raw data was performed using Pilon (parameters: default) [14]. In order to evaluate the completeness and quality of the genome assembly, Benchmarking Universal Single-Copy Orthologs (BUSCO) was used to search for annotated genes in the assembled genome with the vertebrata_odb9 database [15].

### 2.3. Genome Annotation

To identify transposable elements (TEs) and other repetitive elements, we first customized a de novo repeat library of the genome using RepeatModeler [16]. Then, this was combined with the Repbase database to construct the final species-specific repeat library. Finally, the repeat library was imported to RepeatMasker [16] to identify and cluster repetitive elements.

The gene prediction pipeline combined de novo prediction, homology-based prediction, and transcriptome sequence mapping. For the de novo prediction, Augustus [17] and GenScan [18] were employed using appropriate parameters. For the homology-based prediction, the protein sequences of *Gallus*, *Taeniopygia guttata*, *Serinus canaria*, *Parus major*, and *Lonchura striata* obtained from public databases were mapped onto the assembled genome using Genewise [19] to define gene models. In addition, RNA-Seq data from multiple tissues were assembled using Trinity [20]; then, the assembled transcripts were subsequently used as inputs for gene model prediction using PASA [21]. Finally, the genes predicted from the three independent methods were merged into the final consensus of the gene set using EVidenceModeler [22].

The function annotation of the predicted genes was performed by blasting the protein sequences against a number of databases, including nr, Swiss-Prot, eggNOG, InterPro, NR, GO, and KEGG, using an e-value cutoff of 1 × 10^−5^. tRNAs were identified using tRNAscan-SE [23], and rRNAs were determined using RNAmmer [24]. miRNA and snRNAs were identified by searching the genome assembly against the Rfam [25] database using INFERNAL with default parameters (http://infernal.janelia.org/ accessed on 1 April 2021).

### 2.4. Gene Family and Phylogenetic Analysis

Gene clustering was conducted with OrthoMCL [26] by setting the main inflation value to 1.5 and other parameters as default. The orthologous gene families were grouped based on the 12 avian genomes (*Emberiza elegans*, *Gallus*, *Taeniopygia guttata*, *Pseudopodoces humilis*, *Serinus canaria*, *Parus major*, *Lonchura striata*, *Anas platyrhynchos*, *Philomachus pugnax*, *Corvus brachyrhynchos*, *Melopsittacus undulatus*, and *Nipponia nippon*). Then, the expansion and contraction of the orthologous gene families were identified with the BEGFE software [27].

Phylogenetic trees were constructed using single-copy orthologous genes. Sequences of each group of orthologous genes were first aligned with MUSCLE [28], and then maximum-likelihood trees were constructed based on the alignments using the RAxML program [29]. MP-EST was used to combine different gene trees to obtain the final species tree [30]. To estimate the divergence time, we applied the MCMCtree program of PAML [31], with six calibration points as prior settings, which was adopted from Timetree (http://www.timetree.org, accessed on 1 May 2021). The calibration time includes data for *Gallus gallus* and *Melopsittacus undulates* (92.1–104.0 million years ago (Mya)), *Gallus gallus* and *Anas platyrhynchos* (74–86 Mya), *Melopsittacus undulates* and *Nipponia nippon* (77–90 Mya), *Serinus canaria* and *Taeniopygia* guttata (21–47 Mya), *Serinus canaria* and *Parus major* (36–51 Mya), *Philomachus pugnax*, and *Nipponia nippon* (72–86 Mya).

### 2.5. Analysis of Positive Selection

The rate ratio of nonsynonymous to synonymous nucleotide substitutions (Ka/Ks, or ω) among the four closely related passerine species (*S. canaria*, *T. guttata*, *L. striata*, and *E. elegans*) was estimated using the codeml incorporated in the PAML package [31]. The branch-site models were used to identify positively selected genes on the branch of the yellow-throated bunting based on the CDS sequence and corresponding tree topology of each single-copy orthologous gene family. We then performed a likelihood ratio test and identified the positively selected genes by means of FDR adjustment with Q-values < 0.05. Finally, KEGG enrichment analysis was performed on the screened positively selected genes.

### 2.6. Population Dynamics

The software SAMTools [32] was employed to detect single-nucleotide polymorphisms (SNPs) between diploid chromosomes for the yellow-throated bunting. Subsequently, we used the pairwise sequentially Markovian coalescent (PSMC) program [33] to infer the demographic history of this species. We used the estimate of the mutation rate of 2.01 × 10^−9^ per site per year and the generation time of 3.6 years (IUCN Red List).

## 3. Results

### 3.1. De Novo Genome-Sequencing Assembly and Assessment

K-mer analysis was performed using 85.95 Gb of high-quality clean sequences to estimate genome size, GC content, and heterozygosity rate of *E. elegans*, which were ~1.48 Gb, ~45.1%, and ~0.6%, respectively (Table S1). Then, a total of 95.78 Gb data in PromethION sequences with a read N50 of 22,253 bp were used to assemble the bird genome. A de novo assembly yielded a draft genome of 1.14 Gb and a GC content of 42.9%, which contained 4522 contigs with an N50 length of 16.28 Mb and 4026 scaffolds with an N50 length of 28.94 Mb, respectively (Table 1). This result was in the top 15% of contig N50 and scaffold N50 compared with 637 known avian assembled genomes [34].

We evaluated the completeness of the draft genome assembly by calculating coverage for a set of single-copy orthologous genes in Aves using BUSCO [15]. Our gene predictions recovered 97.3% of the highly conserved orthologs in the lineage, with 96.8% classified as “complete and single-copy” and 0.5% as “complete and duplicated”. Additionally, the assessment of the base content also indicated that the GC content of the assembly was 42.91%, which was similar to the values found for other bird species such as *P. humilis* and *T. guttata* [35].

### 3.2. Genome Annotation

**Figure 2:**
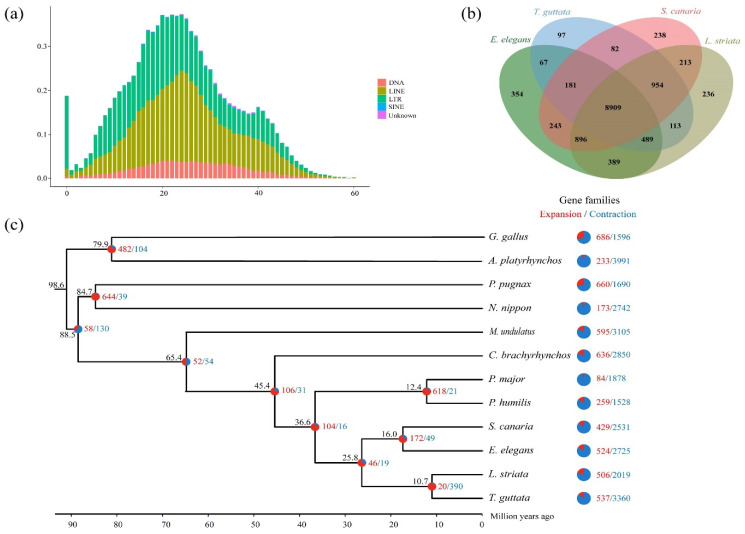
Comparative genomics in avian species studied: (**a**) divergence rate of transposable elements. The x-axis represents the divergence rate of annotated transposable elements, while the y-axis represents the genomic percentage of the corresponding transposable elements; (**b**) Venn diagram showing the number of orthogroups identified within the four Passeriformes species: *E. elegans* (green), *T. guttata* (blue), *S. canaria* (pink), and *L. striata* (brown); (**c**) phylogenetic relationships and divergence times between the 12 avian species estimated from MCMCtree. The numbers to the left of each node indicate divergence time. The expansion and contraction of gene families in these 12 species are shown in different colors, where red represents expanded gene families, and blue represents contracted gene families.

According to the annotation, 16.62% (190 Mb) of the genome assembly was identified as repeats, of which long terminal repeats (LTRs) accounted for the most at 13.26%, followed by long interspersed nuclear elements (LINEs) for 3.52%, DNA transposons for 0.10% and short interspersed nuclear elements (SINEs) for 0.01% (Figure 2a; Table S2).

For the gene prediction and annotation of the assembly, we used a combination of de novo prediction, homology search, and RNA-seq-assisted prediction. The annotation resulted in 15,868 gene models with an average gene length of 25,516  bp and average CDS length of 1600 bp, which share similar gene structures to those of the other published bird genomes (Table 2).

Subsequently, we annotated gene functions based on a known protein database. In total, 15,416 genes were identified with annotated function, accounting for 97.2% of all the genes (Table S3). Additionally, we annotated a total of 1072 noncoding RNAs (ncRNAs) (Table S4), comprising 308 microRNAs (miRNAs), 151 ribosomal RNAs (rRNAs), 284 small-nucleolar RNAs (snRNAs), and 329 transfer RNAs (tRNAs).

### 3.3. Phylogenetic Relationships and Divergence Times

To perform orthologous gene clustering, we used the genes of yellow-throated bunting and 11 other available birds, mentioned above. Gene family clustering identified 14,527 orthologous groups, including 6407 orthologous gene families shared by all species (including 4593 single-copy orthologous gene families) and 8120 nonshared orthologous gene families (Figure S1). We also compared the orthologous gene clusters among four Passeriformes species (*E. elegans*, *T. guttata*, *S. canaria*, and *L. striata*), which showed that 354 orthologous gene families are specific to *E. elegan* (Figure 2b).

The gene trees constructed using each single-copy orthologous gene were merged to form the final species tree using MP-EST (Figure S2). This result well described the phylogenetic relationship between the yellow-throated bunting and 11 other related bird species, and the divergence time was estimated based on the results. The phylogenetic tree showed that *S. canaria* was most closely to *E. elegans* and was grouped into a single branch, with an estimated divergence time of approximately 16 million years ago (Ma). Furthermore, the common ancestor of Passeriformes family members (*E. elegans*, *T. guttata*, *S. canaria*, *L. striata*, *P. major*, *P. humilis*, and *C. brachyrhynchos*) was estimated to have diverged from *M. undulatus* (Psittaciformes) approximately 65.4 Ma (Figure 2c).

### 3.4. Gene Family Expansion and Positive Selection

The results of the expansion and contraction analysis of the gene families showed that *E. elegans* had 524 expanded gene families and 2725 contracted gene families. Its close relative, *S. canaria*, had fewer expanded (429) and contracted (2531) gene families (Figure 2c).

The positive selection analysis identified a total of 1765 positively selected genes in the yellow-throated bunting. The functional enrichment analysis of these genes using KEGG annotations (https://www.genome.jp/kegg/, accessed on 1 April 2021) indicated that they were significantly enriched in 26 pathways (*p*-value < 0.05, Figure S3, Table S5), most of which are related to growth and development, fat metabolism, immune response, and other physiological adaptations.

We found 3 major signaling pathways harboring 41 genes involved in cell proliferation and differentiation (Table S5), namely the MAPK signaling pathway (KO04010), the ErbB signaling pathway (KO04012), and the PI3K–Akt signaling pathway (KO04151). MAPK, ErbB family, and PI3K–Akt play important roles in cell proliferation, differentiation, growth, and survival [36]. At the same time, some other enriched pathways have also shown significant effects on cell growth, such as ribosome biogenesis in eukaryotes (KO03008), which involves the production and correct assembly of ribosome proteins and affects the growth state of cells [36], and focal adhesion (KO04510), which is involved in cell proliferation, cell differentiation, and cell motility [36]. These pathways in combination played important roles in the growth and development of the yellow-throated bunting.

Two signaling pathways referring to thirteen genes (Table S6) were identified that may be related to fat metabolism. Fat digestion and absorption (KO04975) regulate the decomposition and resynthesis of long-chain triglycerides [37], and some genes involved in this pathway, such as *mogat2*, *abcg8*, and *cel*, were all positively selected. The adipocytokine signaling pathway (KO04920) acts on adipocytes and regulates their volume and number [38], and we found *g6pc2*, *ppara*, and other genes in this pathway, which were all positively selected. Additionally, 7 pathways were found containing 40 positively selected genes that may be associated with the immune response of the yellow-throated bunting. For example, the chemokine signaling pathway (KO04062) and the Fc Epsilon RI signaling pathway (KO04664), which act on leukocytes and mast cells, respectively, participate in the regulation of inflammatory immune responses [39,40]. The T-cell (KO04660) and B-cell (KO04662) receptor signaling pathways are involved in the proliferation and differentiation of T cells and B cells, respectively [36]. Among the above-mentioned enriched pathways related to the immune response, *cx3ccr1*, *lpc2*, *blnk*, and other genes involved in regulating inflammatory or autoimmune responses were also all identified under positive selection. In addition, we also found other pathways related to physiological adaptation (Figure S3), such as adrenergic signaling in cardiomyocytes and the oxytocin signaling pathway acting on cardiomyocytes and cardiovascular regulation, respectively [41,42]. Some key genes in the above pathways such as *tnnc1* and *mylk3* were positively selected. Furthermore, it was found that several positively selected genes were associated with circadian rhythms (Table S6). All these positively selected genes may be related to environmental adaptation and migration behavior.

### 3.5. Temporal Population Dynamics

**Figure 3:**
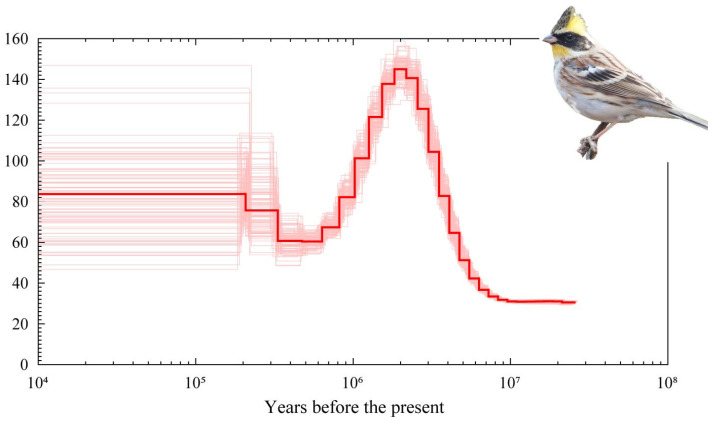
Demographic histories of yellow-throated bunting reconstructed using the PSMC model. The central red bold lines represent inferred population sizes, whereas thin lines are 100 individual bootstrap replicates.

We analyzed the effective population dynamics of yellow-throated bunting using PSMC and observed signs of major cycles of population fluctuation over the past 10 million years (Figure 3). Historical effective population size (N_e_) analyses indicated that the yellow-throated bunting population size experienced two expansions in the past 10 million years (10^7^) but underwent one historical N_e_ decline. From 10 million years ago (Mya) to 3 Mya, the population of yellow-throated bunting experienced the first expansion in its demographic history. A decline in its effective population size began ~2.5 Mya and continued for a while thereafter. However, the population of yellow-throated bunting began to increase slowly from 500,000 years ago, which was then followed by a period of stability for around 200,000 years.

## 4. Discussion

### 4.1. Genomic Features

We assembled a highly contiguous genome assembly for *E. elegans* and produced a high-quality annotation for this species. First, the genome size of the yellow-throated bunting (1.14 Gb) is comparable to other avian genomes [43]. Meanwhile, compared with the conventional Illumina-sequenced bird genomes [35], this assembly showed higher scaffold N50 (28.94 Mb) and larger contig N50 (16.28 Mb). Second, the number and length of the genes obtained from the genome annotation of the yellow-throated bunting were found to be within the range typically expected for birds in general (Table 2). Finally, scans for core genes using the BUSCO databases identified the nearly entire number of these genes in the assembly (97.3%) and support the claim that we largely reconstructed the entire genome. Based on these comparisons, we infer that our assembly represents a nearly complete and high-quality genome of *E. elegans.* This genome will facilitate further research into the evolution of yellow-throated buntings and serve as a high-quality reference genome for population genetic studies.

We noted that this assembly contained a higher proportion of repetitive sequences (16.62%, Figure 2a; Table S2) in comparison with other avian genomes (e.g., *P. humilis* 7%, *T. guttata* 12.06%) [35]. On the one hand, this may be because the third-generation Nanopore long-read sequencing overcomes the limitations of short-read sequencing technologies and can more likely improve the representation of repetitive elements [43]. On the other hand, flying birds usually have higher repeat sequences than flightless birds [44]; hence, the yellow-throated bunting has more proportion of repeats than a long-distance migratory bird.

Furthermore, the proportion of long terminal repeat retrotransposons (LTRs) in the genome of the yellow-throated bunting was found to be higher than that in other avian genomes (Figure 2a, Table S2). First, the long-read sequencing technology used for this bird assembly can restore a more comprehensive and complete LTR structure, which makes its density and diversity relatively higher in the genome [45,46,47]. Second, the expansion of LTR abundance appears to be characteristic of passerine birds [48], which is particularly obvious in yellow-throated buntings. Studies have shown that the abundance and diversity of LTR retrotransposons are consistent with the expansion of the immune gene family [45]. In fact, positively selected genes in the yellow-throated bunting were significantly enriched in several pathways related to immune defense (Figure S3, Table S5), suggesting that there may be an arms race between the immune gene family and LTR. However, the specific LTR density and diversity, as well as its relationship with immune gene families, need to be further explored in follow-up studies.

### 4.2. Functional Assignment

As a songbird, the yellow-throated bunting exhibits migratory traits distinct from those of other songbirds, which may result in its exposure to different environmental selection pressures and is more likely to show different adaptive evolutionary characteristics from other songbirds. In order to understand these features of the yellow-throated bunting, we selected common oscine passerines with high genome assembly quality and no long-distance migration ability (*T. guttata*, *S. canaria*, *L. striata*) for further comparative analysis. The results from the enrichment analysis of positively selected genes suggested that many molecular adaptation mechanisms related to fat metabolism, physiological adaptation, and the immune response may be the consequence to adapt to migratory behavior.

First, migratory birds have extremely high energy consumption during their migration period, as they require a large amount of fatty acid metabolism to maintain long-distance sustained flapping flight [49]. Here, several significantly enriched signaling pathways were found to be implicated in fat metabolism processes, such as fat digestion and absorption, and the adipocytokine signaling pathway (Figure S3, Table S5). Additionally, several positively selected genes directly related to fat metabolism were identified (Table S6), such as PPAR-alpha (*ppara*) and *cel* genes, which are involved in fat uptake and storage [50,51]. The above evidence suggests that the yellow-throated bunting may cope with the high energy consumption during long-distance migratory flight by enhancing its lipid uptake and utilization. 

Second, long-distance migratory birds have evolved a series of physiological adaptations [52]. We found that some positively selected genes were significantly enriched in multiple pathways that may be involved in such adaptations (Figure S3, Table S5). For example, adrenergic signaling in cardiomyocytes and the oxytocin signaling pathway may adapt to the physiological strain resulting from long-distance flying by regulating the cardiac muscles and cardiovascular system of yellow-throated bunting. The thyroid hormone signaling pathway can promote the seasonal migration of migratory birds by regulating thyroid hormones [53]. Moreover, studies have shown that some circadian clock genes are involved in controlling many components of migration behavior in birds, including the timing, extent, and duration of migration [54]. Here, we identified some positively selected genes that are directly involved in the pathway of circadian regulation (Table S6), although this pathway was not significantly enriched. For example, *per3* is a central component of the biological clock and strongly contributes to the regulation of circadian rhythmicity [54]. *skp1* encodes a component of the SCF complex that regulates circadian clock oscillations by targeting Cry protein degradation [55]. All these traits may be the consequences of physiological adaptation to migratory behavior in the yellow-throated bunting. 

Finally, the migratory behavior of birds may also cause the rapid spread of pathogens and parasites [56], which poses a tough challenge to the immune system of birds. In this study, we observed some pathways that are significantly involved in the immune response and pathogen resistance (Figure S3). At the same time, we found some well-known genes involved in the immune response (such as *cx3ccr1*, *lpc2*, *blnk*) in yellow-throated buntings, which were identified in the positive selection results. Based on the above analysis, we believe that the yellow-throated bunting has a fairly strong ability to cope with environmental stressors and diseases, which may be the result of the selection pressure of pathogenic bacteria during its migration.

In summary, the adaptation mechanism of yellow-throated bunting to migration behavior may be reflected in the effective utilization of lipids and a series of physiological adaptations during flight. On the other hand, it may also reflect enhanced disease resistance to pathogens during migration.

### 4.3. Demographic History

Yellow-throated bunting populations have historically experienced cycles of expansion and contraction (Figure 3), which is not unexpected for the Palearctic species during Pleistocene periods of alternating glacials and interglacials. Many birds suffered similar population dynamics during this period, and this seems affected by glacial cycles [57]. Likewise, during the Last Glacial period (LGP), the most pronounced pattern observed in many species was a severe population decline, consistent with the onset of the glacial period [57]. However, we did not observe this phenomenon in yellow-throated buntings; in contrast, their populations were able to remain stable even under the Last Glacial Maximum (LGM). We speculated that the difference could be attributed to two reasons. First, yellow-throated buntings inherited their migration trait from their ancestors [6], which can help them find more suitable habitats and refuges, such as southern China, the Dabie Mountains, the Hengduan Mountains, etc. [58,59], thereby reducing the impact of climate change on the population over the whole period of the LGP. Second, since the population size of many species drastically decreased over the same period [59], the yellow-throated bunting with strong adaptability can obtain more resources and space to achieve population stability. Overall, it is possible that the yellow-throated bunting experienced adaptation and dealt with the glacial climate changes, ultimately resulting in the populations being the most climatically tolerant during the LGM.

## 5. Conclusions

In this study, we presented the de novo assembled high-quality genome of the yellow-throated bunting and compared it with other avian genomes. Our genomic evolution analyses found considerable numbers of adaptive genes that may help the yellow-throated bunting cope with migratory behavior and environmental stressors of diseases. These results provide us with new insights into genomic evolution and adaptation, and it will be a very important resource for investigations associated with the adaptive evolution of migration behavior, comparative genomics of other avian species, and population genetic diversity of the yellow-throated bunting.

## Figures and Tables

**Table 1:** Assembly statistics of the yellow-throated bunting genome.

	Contig	Scaffold
Number	4522	4026
N50	16,287,717	28,938,815
N90	1,224,325	2,340,365
Total length	1,141,058,764	1,144,605,635

**Table 2:** Gene structure annotation statistics and other avian genome comparisons.

Species	Number	Average Transcript Length (bp)	Average CDS Length (bp)	Average Exons per Gene	Average Exon Length (bp)	Average Intron Length (bp)
*Emberiza elegans*	15,868	25,516.83	1600.26	9.62	166.30	2773.68
*Gallus gallus*	17,444	29,696.28	1735.32	10.19	170.35	3043.54
*Lonchura striata*	15,420	28,472.72	1774.93	10.66	166.45	2762.73
*Parus major*	15,240	28,701.65	1802.84	10.76	167.49	2754.87
*Serinus canaria*	14,756	27,514.15	1729.92	10.74	161.08	2647.35
*Taeniopygia guttata*	16,351	26,215.51	1619.92	10.01	161.88	2730.73

## Data Availability

The dataset used for this study has been deposited in GenBank under BioProject PRJNA825116/BioSample accession SAMN27502735. This Whole-Genome Shotgun project has been deposited at DDBJ/ENA/GenBank under the accession JALJVW000000000. The sequencing data were deposited under NCBI accession PRJNA859540-PRJNA 859544. All other data supporting the findings of this study are available from the corresponding author on reasonable request.

## Supplementary Materials

The following supporting information can be downloaded at: https://www.mdpi.com/article/10.3390/ani12152004/s1. Figure S1: The number of different orthologous genes within the 12 avian species. X-axis denotes species, while the Y-axis indicates the number of genes. The single-copy orthologous, multiple-copy orthologous, species-specific orthologous, and other orthologous of each species are represented by red, orange, brown, and green, respectively; Figure S2: Species trees of 12 birds were constructed using maximum-likelihood trees of each single-copy orthologous gene family using MP-EST; Figure S3: Horizontal bar plots represent significantly enriched positively selected gene pathway types and gene numbers. The horizontal axis is the number of genes, and the vertical axis is the pathway’s name; Table S1: Genomic characteristics statistics of *E. elegans* obtained by using K-mer analysis; Table S2: Repetitive element statistics; Table S3: Statistical analysis of functional annotated protein-coding genes; Table S4: Statistical results of non-coding RNAs; Table S5: Positively selected genes’ significantly enriched pathways in KEGG enrichment analysis; Table S6: Positively selected genes associated with circadian rhythms and fat metabolism from KEGG enrichment analysis.

## References

[1] MacKinnon J. (2022). Guide to the Birds of China.

[2] Zheng G.M. (2002). A Checklist on the Classification and Distribution of the Birds of China.

[3] Nam H.Y., Choi C.Y., Park J.G., Hong G.P., Won I.J., Kim S.J., Bing G.C., Chae H.Y. (2011). Protandrous migration and variation in morpho-logical characters in Emberiza buntings at an East Asian stopover site. Ibis.

[4] Cai T., Wu G., Sun L., Zhang Y., Peng Z., Guo Y., Liu X., Pan T., Chang J., Sun Z. (2021). Biogeography and diversification of Old World buntings (*Aves: Emberizidae*): Radiation in open habitats. J. Avian Biol..

[5] Wang S.R., Joka F.R., Wang X.L., Bai S.Y. (2019). Analysis of the Phylogeny and Evolutionary Selection Pressure of the Mx Gene in 10 Wild Birds. Pak. J. Zool..

[6] Wang Y.H., Wu W., An Y.X., Zhang W., Xu Q. (2019). Protandry in Yellow-Throated Bunting (*Emberiza elegans*) During Spring in the Maoershan Mountain region of Heilongjiang Province, China. Chin. J. Wildl..

[7] Bennetzen J.L. (2007). Patterns in grass genome evolution. Curr. Opin. Plant Biol..

[8] Stiller J., Zhang G. (2019). Comparative Phylogenomics, a Stepping Stone for Bird Biodiversity Studies. Diversity.

[9] Marçais G., Kingsford C. (2011). A fast, lock-free approach for efficient parallel counting of occurrences of k-mers. Bioinformatics.

[10] Liu B.H., Shi Y.J., Yuan J.Y., Hu X.S., Zhang H., Li N., Li Z.Y., Chen Y.X., Mu D.S., Fan W. (2013). Estimation of genomic characteristics by analyzing k-mer frequency in de novo genome projects. arXiv.

[11] Koren S., Walenz B.P., Berlin K., Miller J.R., Bergman N.H., Phillippy A.M. (2017). Canu: Scalable and accurate long-read assembly via adaptive *k*-mer weighting and repeat separation. Genome Res..

[12] Vaser R., Sović I., Nagarajan N., Šikić M. (2017). Fast and accurate de novo genome assembly from long uncorrected reads. Genome Res..

[13] English A.C., Richards S., Han Y., Wang M., Vee V., Qu J., Qin X., Muzny D.M., Reid J.G., Worley K.C. (2012). Mind the Gap: Upgrading Genomes with Pacific Biosciences RS Long-Read Sequencing Technology. PLoS ONE.

[14] Walker B.J., Abeel T., Shea T., Priest M., Abouelliel A., Sakthikumar S., Cuomo C.A., Zeng Q., Wortman J., Young S.K. (2014). Pilon: An Integrated Tool for Comprehensive Microbial Variant Detection and Genome Assembly Improvement. PLoS ONE.

[15] Simão F.A., Waterhouse R.M., Ioannidis P., Kriventseva E.V., Zdobnov E.M. (2015). BUSCO: Assessing genome assembly and annotation completeness with single-copy orthologs. Bioinformatics.

[16] Tarailo G.M., Chen N. (2009). Using RepeatMasker to identify repetitive elements in genomic sequences. Curr. Protoc. Bioin-Form..

[17] Stanke M., Keller O., Gunduz I., Hayes A., Waack S., Morgenstern B. (2006). AUGUSTUS: Ab initio prediction of alternative transcripts. Nucleic Acids Res..

[18] Burge C., Karlin S. (1997). Prediction of complete gene structures in human genomic DNA1. J. Mol. Biol..

[19] Birney E., Durbin R. (2000). Using GeneWise in the Drosophila annotation experiment. Genome Res..

[20] Grabherr M.G., Haas B.J., Yassour M., Levin J.Z., Thompson D.A., Amit I., Adiconis X., Fan L., Raychowdhury R., Zeng Q.D. (2011). Full-length transcriptome assembly from RNA-Seq data without a reference genome. Nat. Biotechnol..

[21] Haas B.J., Delcher A.L., Mount S.M., Wortman J.R., Smith R.K., Hannick L.I., Maiti R., Ronning C.M., Rusch D.B., Town C.D. (2003). Improving the Arabidopsis genome annotation using maximal transcript alignment assemblies. Nucleic Acids Res..

[22] Haas B.J., Salzberg S.L., Zhu W., Pertea M., Allen J.E., Orvis J., White O., Buell C.R., Wortman J.R. (2008). Automated eukaryotic gene structure annotation using EVidenceModeler and the Program to Assemble Spliced Alignments. Genome Biol..

[23] Lowe T.M., Eddy S.R. (1997). tRNAscan-SE: A program for improved detection of transfer RNA genes in genomic sequence. Nucleic Acids Res..

[24] Lagesen K., Hallin P., Rodland E.A., Staerfeldt H.H., Rognes T., Ussery D.W. (2007). RNAmmer: Consistent and rapid annotation of ri-bosomal RNA genes. Nucleic Acids Res..

[25] Griffiths J.S., Moxon S., Marshall M., Khanna A., Eddy S.R., Bateman A. (2005). Rfam: Annotating non-coding RNAs in complete genomes. Nucleic Acids Res..

[26] Li L., Stoeckert C.J., Roos D.S. (2003). OrthoMCL: Identification of Ortholog Groups for Eukaryotic Genomes. Genome Res..

[27] Liu L., Yu L., Kalavacharla V., Liu Z. (2011). A Bayesian model for gene family evolution. BMC Bioinform..

[28] Edgar R.C. (2004). MUSCLE: Multiple sequence alignment with high accuracy and high throughput. Nucleic Acids Res..

[29] Stamatakis A. (2006). RaxML-VI-HPC: Maximum likelihood-based phylogenetic analyses with thousands of taxa and mixed models. Bioinformatics.

[30] Liu L., Yu L., Edwards S.V. (2010). A maximum pseudo-likelihood approach for estimating species trees under the coalescent model. BMC Evol. Biol..

[31] Yang Z. (2007). PAML 4: Phylogenetic Analysis by Maximum Likelihood. Mol. Biol. Evol..

[32] Li J., Ng E.K., Ng Y.P., Wong C.Y., Yu J., Jin H., Cheng V.Y., Go M.Y., Cheung P.K., Ebert M.P. (2009). Identification of retinoic ac-id-regulated nuclear matrix-associated protein as a novel regulator of gastric cancer. Br. J. Cancer.

[33] Li H., Durbin R. (2011). Inference of human population history from individual whole-genome sequences. Nature.

[34] Bravo G.A., Schmitt C.J., Edwards S.V. (2021). What have we learned from the first 500 avian genomes?. Annu. Rev. Ecol. Evol. Syst..

[35] Cai Q.L., Qian X.J., Lang Y.S., Luo Y.D., Xu J.H., Pan S.K., Hui Y.Y., Gou C.Y., Cai Y., Hao M.R. (2013). Genome sequence of ground tit Pseu-dopodoces humilis and its adaptation to high altitude. Genome Biol..

[36] Zhai Z.H., Wang X.Z., Ding M.X. (2011). Cell Biology.

[37] Niot I., Poirier H., Tran T.T.T., Besnard P. (2009). Intestinal absorption of long-chain fatty acids: Evidence and uncertainties. Prog. Lipid Res..

[38] Yamauchi T., Kamon J., Minokoshi Y., Ito Y., Waki H., Uchida S., Yamashita S., Noda M., Kita S., Ueki K. (2002). Adiponectin stimulates glucose utilization and fatty-acid oxidation by activating AMP-activated protein kinase. Nat. Med..

[39] Wong M.M., Fish E.N. (2002). Chemokines: Attractive mediators of the immune response. Semin. Immunol..

[40] Gilfillan A.M., Tkaczyk C. (2006). Integrated signalling pathways for mast-cell activation. Nat. Rev. Immunol..

[41] Bers D.M. (2002). Cardiac excitation-contraction coupling. Nature.

[42] Gutkowska J., Jankowski M., Lambert C., Mukaddam-Daher S., Zingg H.H., McCann S.M. (1997). Oxytocin releases atrial natriuretic peptide by combining with oxytocin receptors in the heart. Proc. Natl. Acad. Sci. USA.

[43] Kapusta A., Suh A., Feschotte C. (2017). Dynamics of genome size evolution in birds and mammals. Proc. Natl. Acad. Sci. USA.

[44] Warren W.C., Clayton D.F., Ellegren H., Arnold A.P., Hillier L.W., Künstner A., Searle S., White S., Vilella A., Fairley S. (2010). The genome of a songbird. Nature.

[45] Kapusta A., Suh A. (2016). Evolution of bird genomes-a transposon’s-eye view. Ann. N. Y. Acad. Sci..

[46] Kapusta A., Suh A., Feschotte C. (2016). The hidden elasticity of avian and mammalian genomes. bioRxiv.

[47] Boman J., Frankl-Vilches C., dos Santos M.D., de Oliveira E.H.C., Gahr M., Suh A. (2019). The genome of Blue-Capped Cordon-Bleu Un-covers hidden diversity of LTR retrotransposons in Zebra Finch. Genes.

[48] Zhang G., Li B., Li C., Gilbert M.T.P., Jarvis E.D., Wang J., The Avian Genome Consortium (2014). Comparative genomic data of the Avian Phylogenomics Project. GigaScience.

[49] Guglielmo C.G., Haunerland N.H., Hochachka P.W., Williams T.D. (2002). Seasonal dynamics of flight muscle fatty acid binding protein and catabolic enzymes in a migratory shorebird. Am. J. Physiol. Integr. Comp. Physiol..

[50] Corder K.R., DeMoranville K.J., Russell D.E., Huss J.M., Schaeffer P.J. (2016). Annual life-stage regulation of lipid metabolism and storage and association with PPARs in the migrant species Gray Catbird (*Dumetella carolinensis*). J. Exp. Biol..

[51] Wang X., Wang X., Chen B., Guo Y., Tang H., Li D., Liu D., Wang Y., Li G., Kang X. (2020). Association of a new 99-bp indel of the CEL gene promoter region with phenotypic traits in chickens. Sci. Rep..

[52] Wu L., Jiao X., Zhang D., Cheng Y., Song G., Qu Y., Lei F. (2021). Comparative Genomics and Evolution of Avian Specialized Traits. Curr. Genom..

[53] Johnston R.A., Paxton K.L., Moore F.R., Wayne R.K., Smith T.B. (2016). Seasonal gene expression in a migratory songbird. Mol. Ecol..

[54] Kumar V., Wingfield J.C., Dawson A., Ramenofsky M., Rani S., Bartell P. (2010). Biological Clocks and Regulation of Seasonal Reproduction and Migration in Birds. Physiol. Biochem. Zool..

[55] Yumimoto K., Muneoka T., Tsuboi T., Nakayama K.I. (2013). Substrate Binding Promotes Formation of the Skp1-Cul1-Fbxl3 (SCFFbxl3) Protein Complex. J. Biol. Chem..

[56] Figuerola J., Green A.J. (2000). Haematozoan Parasites and Migratory Behaviour in Waterfowl. Evol. Ecol..

[57] Nadachowska-Brzyska K., Li C., Smeds L., Zhang G., Ellegren H. (2015). Temporal Dynamics of Avian Populations during Pleistocene Revealed by Whole-Genome Sequences. Curr. Biol..

[58] Pan T., Wang H., Orozcoterwengel P., Hu C.-C., Wu G.-Y., Qian L.-F., Sun Z.-L., Shi W.-B., Yan P., Wu X.-B. (2019). Long-term sky islands generate highly divergent lineages of a narrowly distributed stream salamander (*Pachyhynobius shangchengensis*) in mid-latitude mountains of East Asia. BMC Evol. Biol..

[59] McCormack J., Huang H., Knowle L., Gillespie R.G., Clague D.A. (2009). Sky islands. Encyclopedia of Islands.

