# Supplementary material for: De Novo Whole-Genome Sequencing and Assembly of the Yellow-Throated Bunting (Emberiza elegans) Provides Insights into Its Evolutionary Adaptation
Source: Animals (Basel). 2022 Aug 8;12(15):2004. doi: 10.3390/ani12152004 (PMC9367368; doi:10.3390/ani12152004)
Supplement: Supplementary file 1 [file animals-12-02004-s001.zip › animals-1716400-supplementary.pdf]

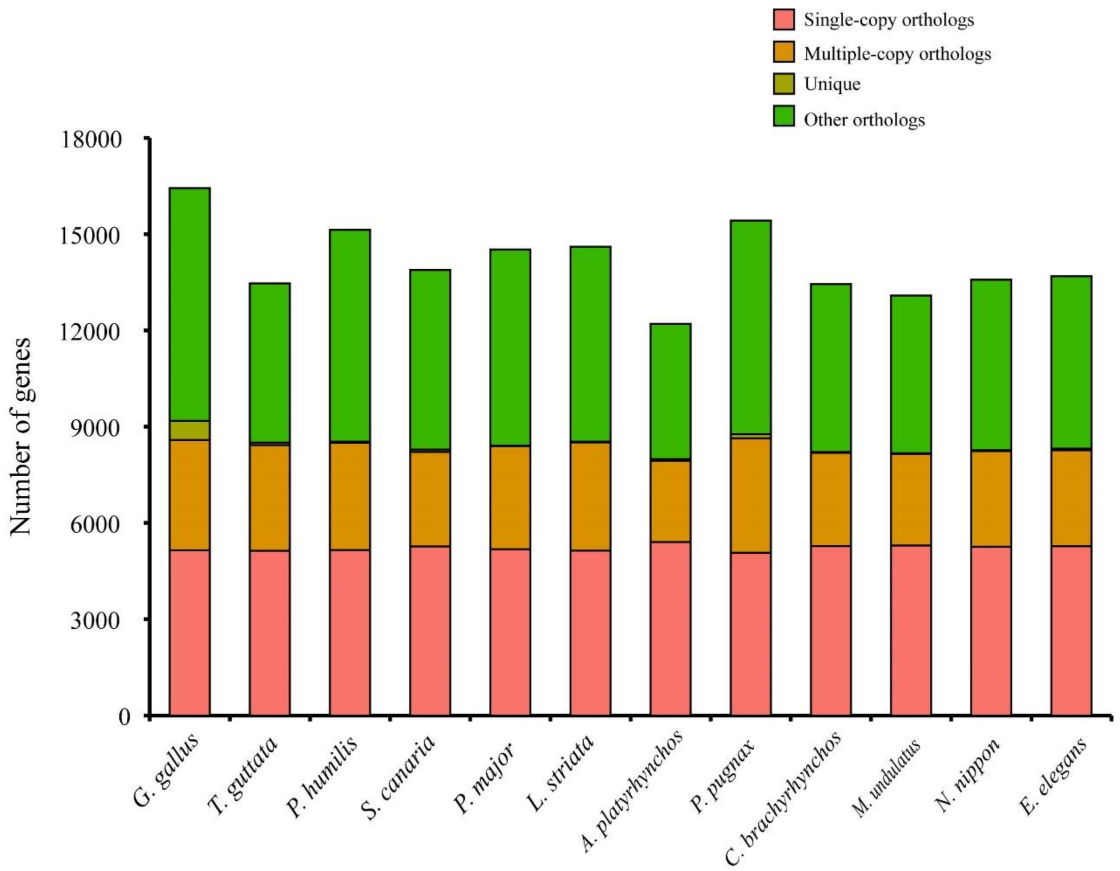

Figure S1. The number of different orthologous genes within the 12 avian species. X-axis denotes species, while the Y-axis indicates the number of genes. The single-copy orthologous, multiple-copy orthologous, species-specific orthologous, and other orthologous of each species are represented by red, orange, brown, and green, respectively.

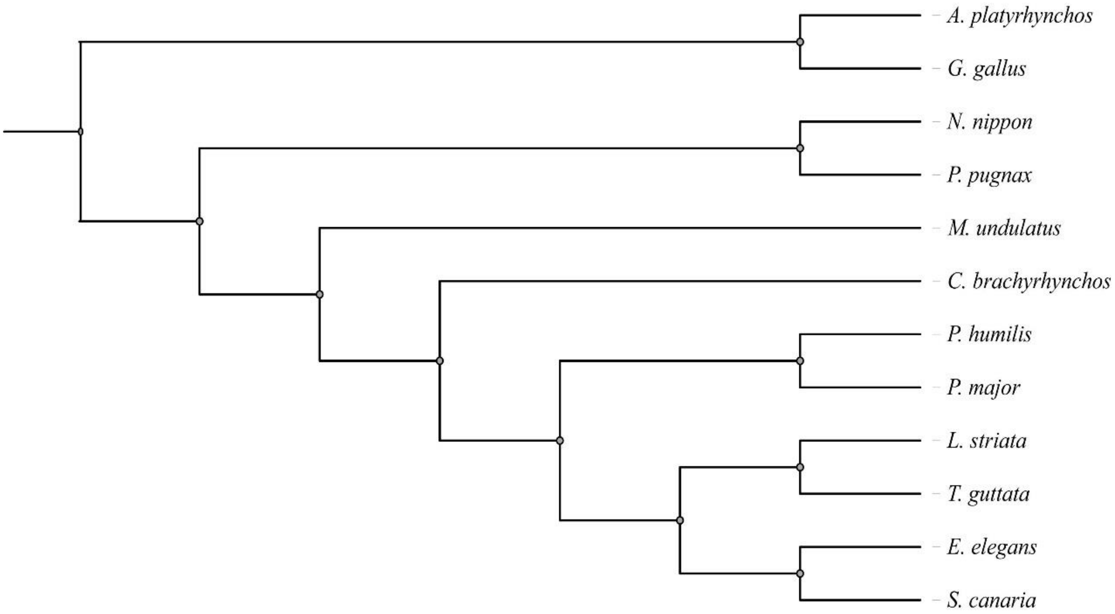

Figure S2. Species trees of 12 birds were constructed using maximum-likelihood trees of each single-copy orthologous gene family using MP-EST.

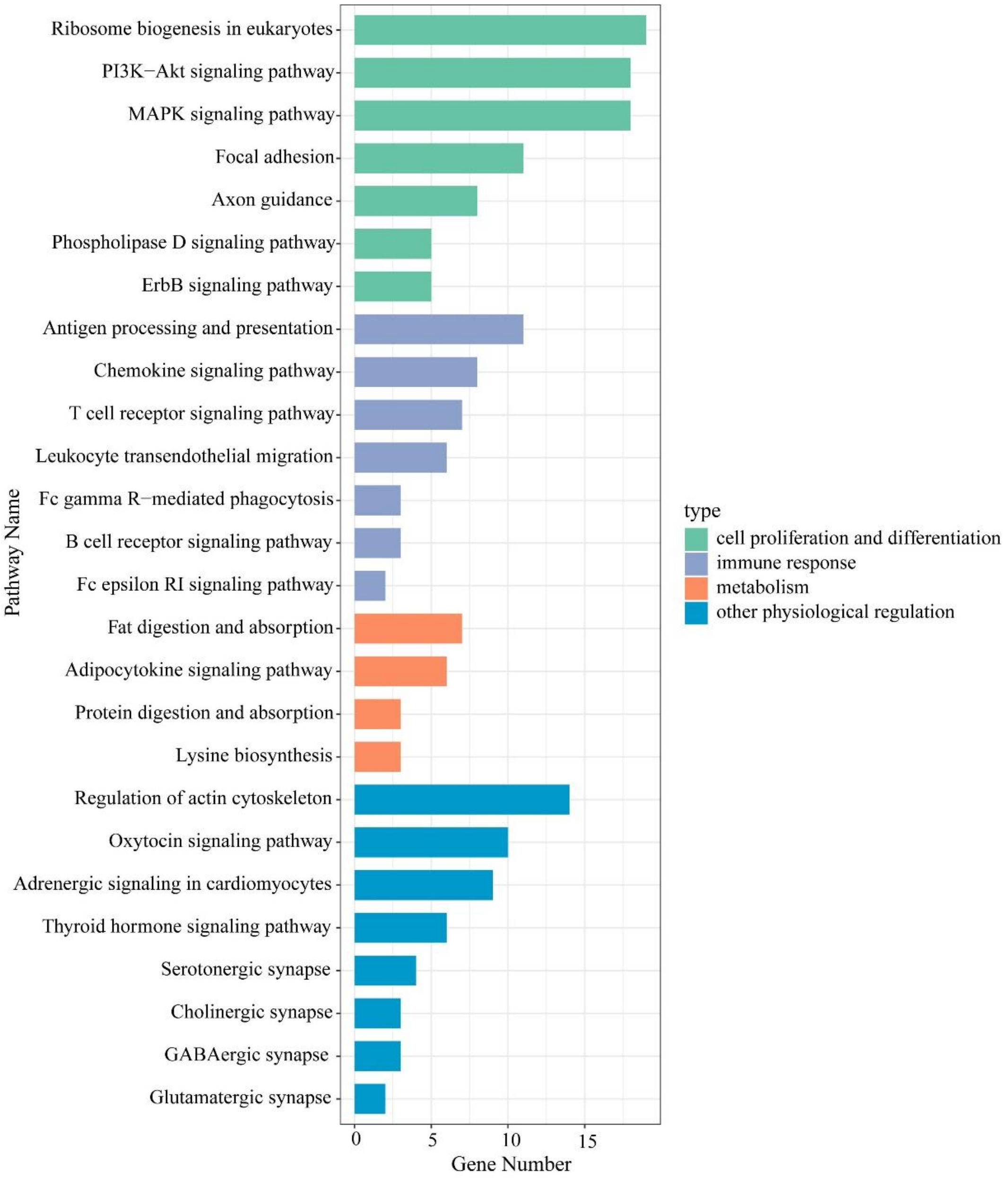

Figure S3. Horizontal bar plots represent significantly enriched positively selected gene pathway types and gene numbers. The horizontal axis is the number of genes, and the vertical axis is the pathway's name.

Table S1. Genomic characteristics statistics of *E. elegans* obtained by using K-mer analysis.

K-mer	K-mer number	K-mer Depth	Genome Size （Mbp）	Revised Genome Size (Mbp)	Heterozygous Ratio (%)	Repeat (%)
17	62,559,975,660	42	1,489.52	1,486.61	0.66	47.81

Table S2. Repetitive element statistics.

Type	Length (bp)	Percentage (%)
DNAs	1,112,875	0.10
LINEs	40,322,527	3.52
SINEs	97,924	0.01
LTRs	151,768,505	13.26
Unclassified	9,606,478	0.84
Total	190,253,193	16.62

Note: Abbreviations indicate DNA transposons (DNAs), long interspersed nuclear elements (LINEs), short interspersed nuclear elements (SINEs), and long terminal repeats (LTRs).

Table S3. Statistical analysis of functional annotated protein-coding genes.

Type		Number	Percent (%)
Annotation	KEGG	13,482	85.0
	GO	10,979	69.2
	KOG	14,696	92.6
	SwissProt	14,945	94.2
	InterPro	14,891	93.8
	NR	15,382	96.9
Total	Annotated genes	15,416	97.2
	Total Genes	15,868	-

Note: "Annotation" is the annotation results of each database and the proportion of annotation coverage. "Annotated genes" represent non-redundant results based on annotations. "Total Genes" is the total number of protein-coding genes (annotated genes + unannotated genes).

Table S4. Statistical results of non-coding RNAs.

Type	Copy (w*)	Average length (bp)	Total length (bp)	Percentage (%)
miRNA	308	89.27	27,495	0.002402
tRNA	329	75.18	24,735	0.002161
rRNA	151	269.78	40,737	0.003559
snRNA	284	129.94	36,904	0.003224

Note: Abbreviations indicate transfer RNA (tRNA), ribosomal RNA (rRNA), small nuclear RNA (snRNA), and microRNA (miRNA).

Table S5. Positively selected genes' significantly enriched pathways in KEGG enrichment analysis.

KO number	Pathway name	Gene number	P-value	Corrected P-value
KO04360	Axon guidance	8	2.00E-04	4.20E-02
KO04510	Focal adhesion	11	4.18E-04	4.28E-02
KO03008	Ribosome biogenesis in eukaryotes	19	5.09E-04	4.29E-02
KO04151	PI3K-Akt signaling pathway	18	8.59E-04	4.29E-02
KO04724	Glutamatergic synapse	2	9.68E-04	4.29E-02
KO00300	Lysine biosynthesis	3	9.91E-04	4.29E-02
KO04072	Phospholipase D signaling pathway	5	2.01E-03	7.64E-02
KO04810	Regulation of actin cytoskeleton	14	4.28E-03	0.1181
KO04725	Cholinergic synapse	3	5.16E-03	0.1302
KO04974	Protein digestion and absorption	3	9.80E-03	0.1820
KO04062	Chemokine signaling pathway	8	0.0114	0.1820
KO04612	Antigen processing and presentation	11	0.0166	0.2511
KO04921	Oxytocin signaling pathway	10	0.0186	0.2575
KO04012	ErbB signaling pathway	5	0.0187	0.2575
KO04726	Serotonergic synapse	4	0.0217	0.2738
KO04010	MAPK signaling pathway	18	0.0301	0.2868
KO04660	T cell receptor signaling pathway	7	0.0310	0.2868
KO04664	Fc epsilon RI signaling pathway	2	0.0312	0.2868
KO04727	GABAergic synapse	3	0.0312	0.2868
KO04666	Fc gamma R-mediated phagocytosis	3	0.0312	0.2868
KO04670	Leukocyte transendothelial migration	6	0.0390	0.3378
KO04662	B cell receptor signaling pathway	3	0.0417	0.3417
KO04662	Thyroid hormone signaling pathway	6	0.0433	0.3417
KO04662	Adrenergic signaling in cardiomyocytes	9	0.0488	0.3417
KO04975	Fat digestion and absorption	7	0.0462	0.3417
KO04920	Adipocytokine signaling pathway	6	0.0488	0.3417

Table S6. Positively selected genes associated with circadian rhythms and fat metabolism from KEGG enrichment analysis.

Function	Gene name	Pathway name (KO number)	Gene ID
Circadian rhythm	PER3	Circadian rhythm (KO04710)	evm.model.FRAGSCAFF_SCAFFOLD_66.166
	SKP1	Circadian rhythm (KO04710)	evm.model.ORIGINAL_SCAFFOLD_803.36.1
	PRKAG2	Circadian rhythm (KO04710)	evm.model.FRAGSCAFF_SCAFFOLD_205.172.1
Fat metabolism	MOGAT2	Fat digestion and absorption (KO04975)	evm.model.FRAGSCAFF_SCAFFOLD_195.124
	PLA2G10	Fat digestion and absorption (KO04975)	evm.model.FRAGSCAFF_SCAFFOLD_242.142
	ABCG8	Fat digestion and absorption (KO04975)	evm.model.ORIGINAL_SCAFFOLD_2636.347
	CEL	Fat digestion and absorption (KO04975)	evm.model.FRAGSCAFF_SCAFFOLD_183.2355
	PLA2G3	Fat digestion and absorption (KO04975)	evm.model.ORIGINAL_SCAFFOLD_3106.270.1
	PLA2G5	Fat digestion and absorption (KO04975)	evm.model.FRAGSCAFF_SCAFFOLD_66.297
	SCARB1	Fat digestion and absorption (KO04975)	evm.model.FRAGSCAFF_SCAFFOLD_183.1779
	G6PC2	Adipocytokine signaling pathway (KO04920)	evm.model.ORIGINAL_SCAFFOLD_3574.260
	RSPH10B	Adipocytokine signaling pathway (KO04920)	evm.model.FRAGSCAFF_SCAFFOLD_196.301
	CIDEA	Adipocytokine signaling pathway (KO04920)	evm.model.ORIGINAL_SCAFFOLD_1430.189
	POMC	Adipocytokine signaling pathway (KO04920)	evm.model.FRAGSCAFF_SCAFFOLD_101.79
	C1QTNF3	Adipocytokine signaling pathway (KO04920)	evm.model.ORIGINAL_SCAFFOLD_2528.122

	PPARA	Adipocytokine signaling pathway (KO04920)	evm.model.FRAGSCAFF_SCAFFOLD_183.929
